# Supplementary figures and images for: Widespread regulation of the maternal transcriptome by Nanos in Drosophila
Source: PLoS Biol. 2024 Oct 14;22(10):e3002840. doi: 10.1371/journal.pbio.3002840 (PMC11501031; doi:10.1371/journal.pbio.3002840)

w Upf1-  
Nos Nos

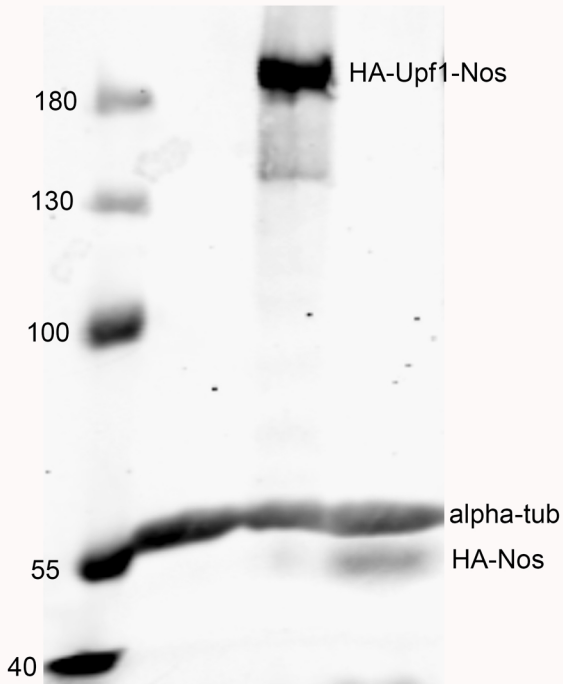

Supplement: S1 Fig — Western blot showing expression of HA-tagged Upf1-Nos and HA-tagged Nos in 0–2-hour embryos. Quantitation is in S1 Data. The first lane is from nontransgenic w embryos and the loading control is alpha-tubulin. MW markers in kDa to the left. Underlying data are in S1 Data. (PDF) [file pbio.3002840.s001.pdf]

MDS Plot – Gene level

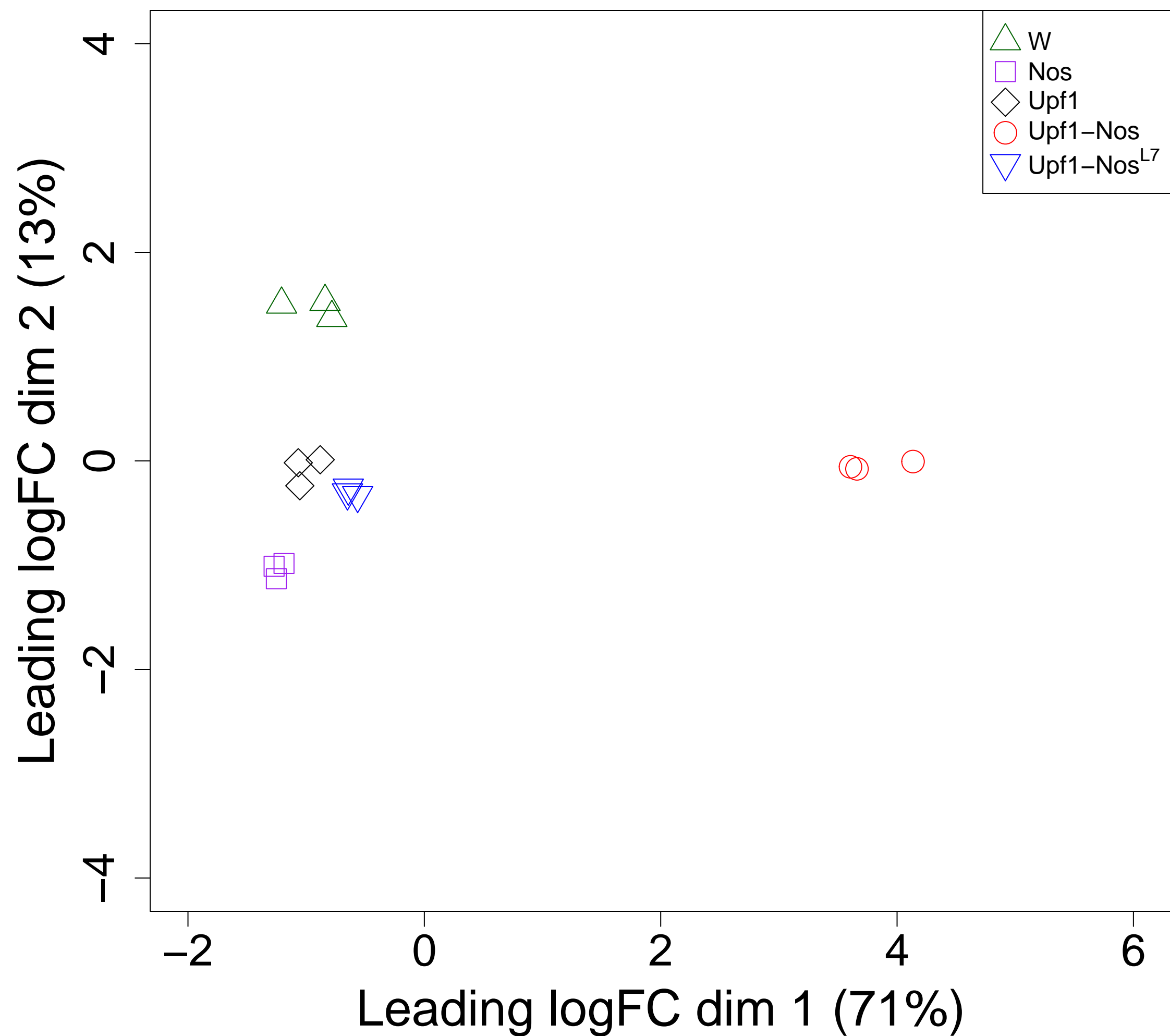

Supplement: S2 Fig — Multidimensional scaling plot of RNAseq data in Fig 2. Underlying data are in S2 Data. (PDF) [file pbio.3002840.s002.pdf]

# Transcript-Level Analysis

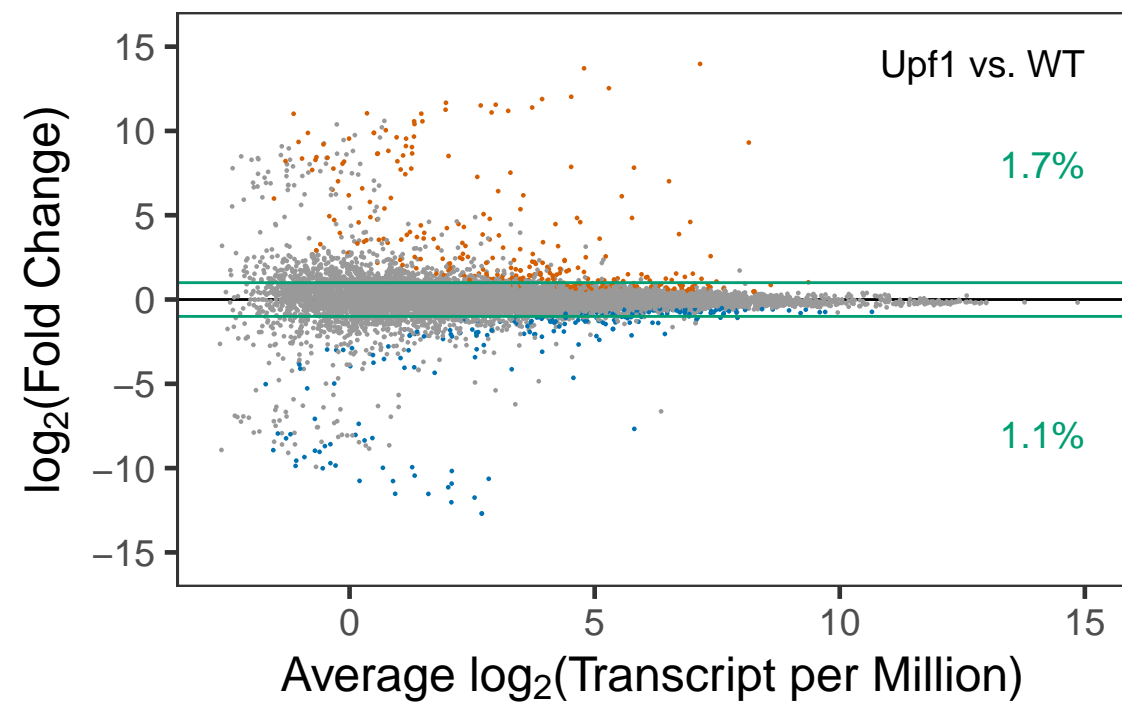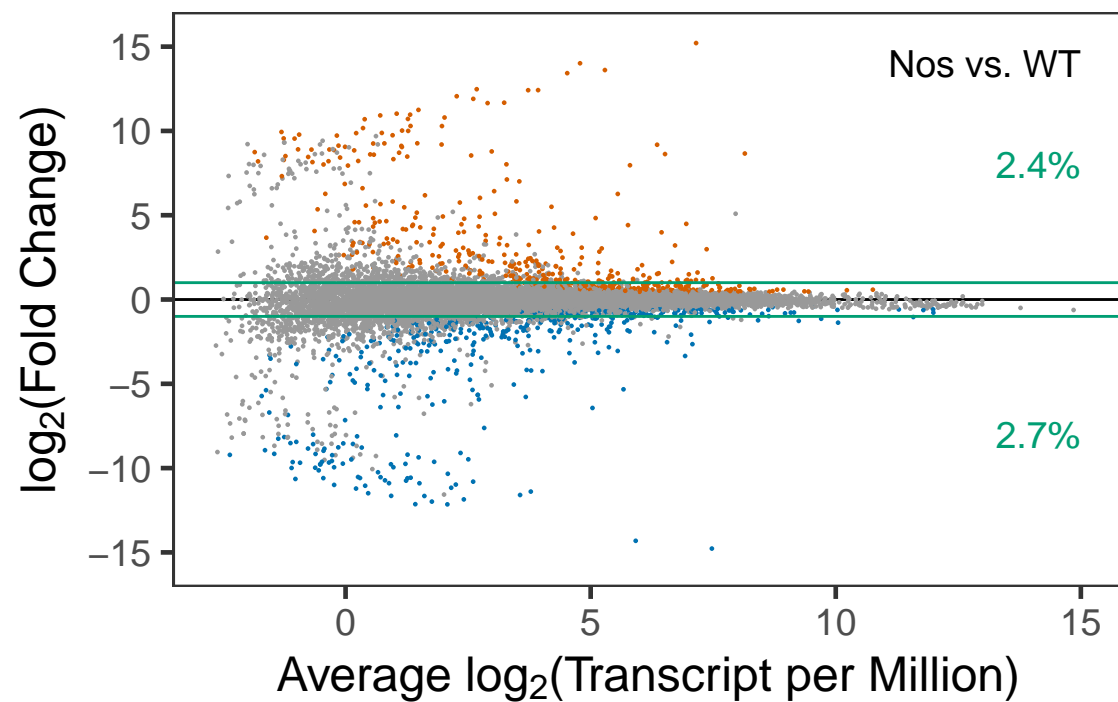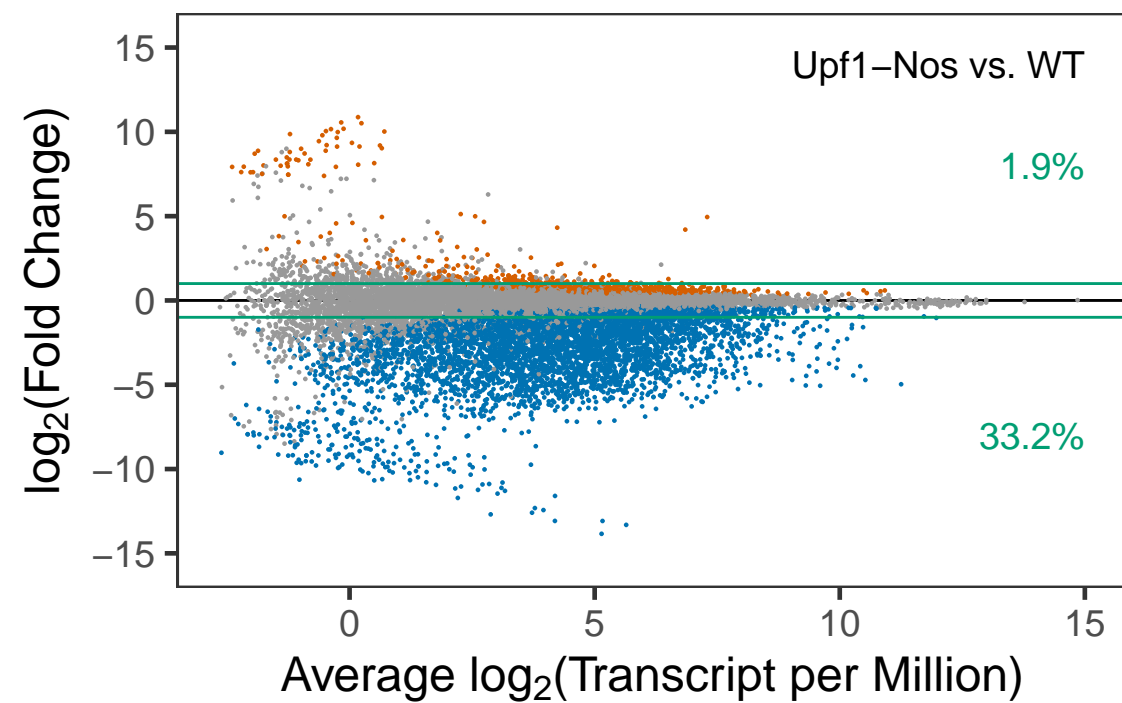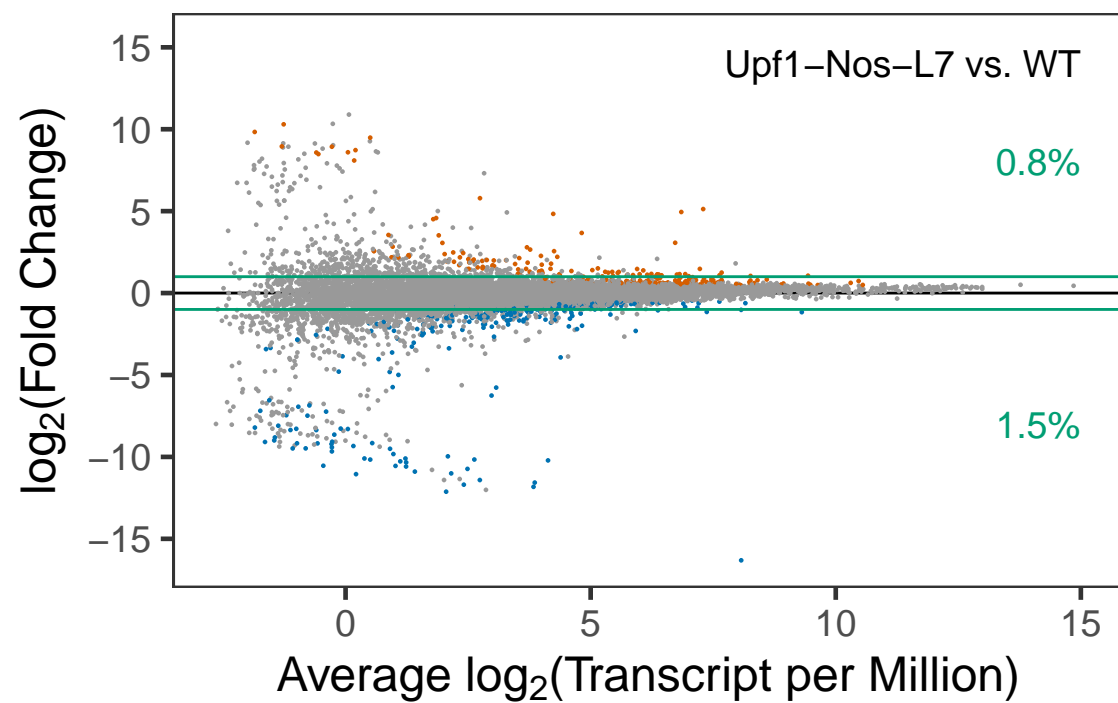

Supplement: S3 Fig — Smear plots of the RNAseq data plotted for individual mRNAs rather than gene-binned mRNAs (as in Fig 2A). Underlying data are in S2 Data. (PDF) [file pbio.3002840.s003.pdf]

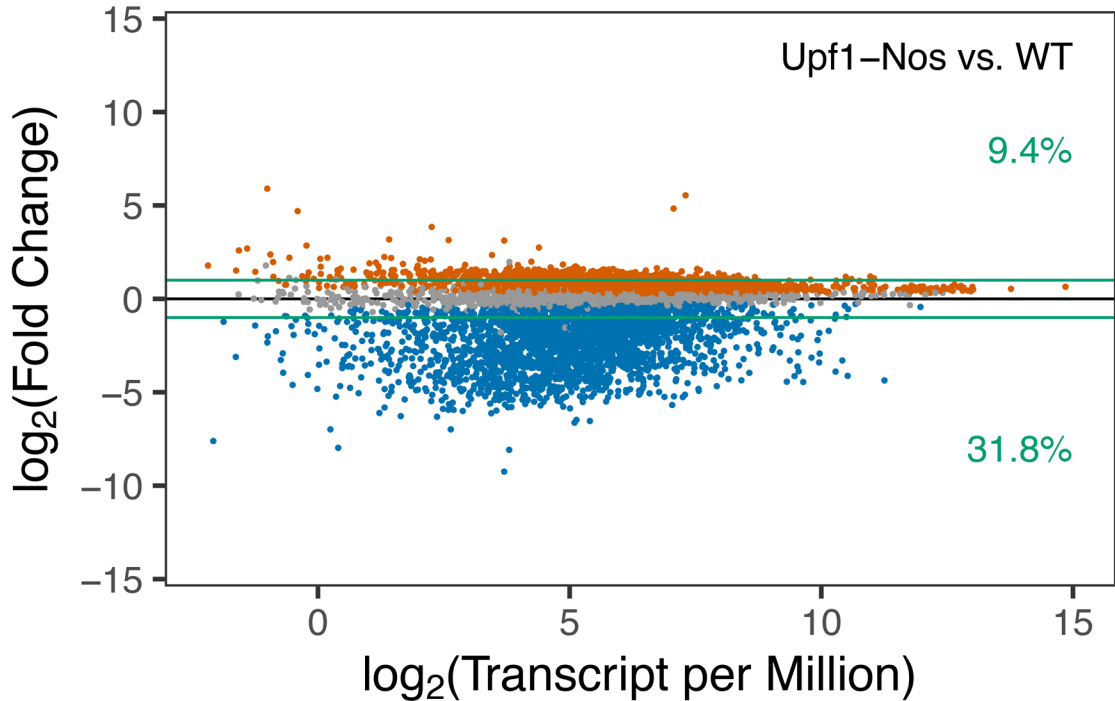

Supplement: S4 Fig — Smear plot displaying relative mRNA levels in Upf1-Nos vs. wt without adjustment for oversampling of unregulated mRNAs. As in Fig 2, the fractions of mRNAs significantly up- and down-regulated are in green to the right. Underlying data are in S2 Data. (PDF) [file pbio.3002840.s004.pdf]

# Upf1-Nos

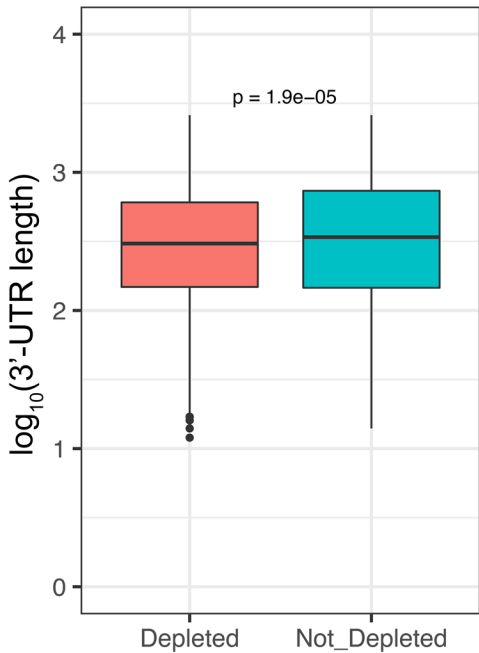

Supplement: S5 Fig — Boxplot showing that mRNAs targeted by Upf1-Nos have, on average, slightly shorter 3′ UTRs than nontargeted mRNAs. Underlying data are in S2 Data. (PDF) [file pbio.3002840.s005.pdf]

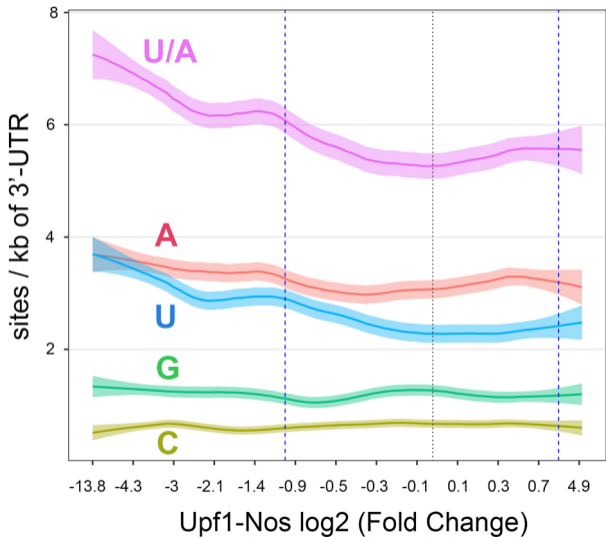

Supplement: S6 Fig — Localized regression of the data analyzed in Fig 3C. Figure shows the density of NRE variants at position +4 in 3′ UTR sequence plotted against the log2 (fold change) in Upf1-Nos vs. wt. Each curve is labelled by the identity of the nucleotide at position +4 in the NRE (i.e., “U/A” corresponds to UAUAUA UGU UA). Here and in the LOESS analyses of Figs 5C and S7, the x-axis is nonlinear, light shading marks 95% confidence windows, the light dashed line marks log2 = 0, and the heavier dashed line marks log2 ± 1. Underlying data are in S2 Data. (PDF) [file pbio.3002840.s006.pdf]

UGUGUGU  
AUAUAU

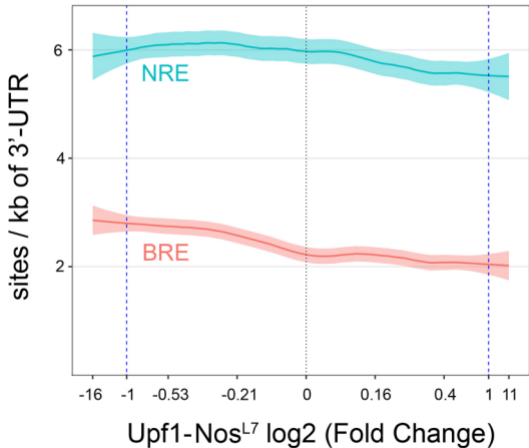

Supplement: S7 Fig — Localized regression of the data analyzed in Fig 4A. Underlying data are in S2 Data. (PDF) [file pbio.3002840.s007.pdf]

# Bru-repressed mRNAs

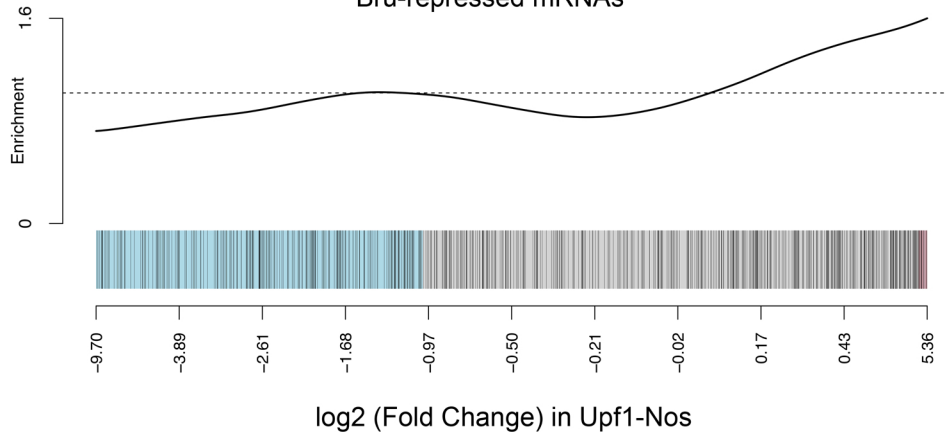

Supplement: S8 Fig — Barcode plot showing no significant enrichment of mRNAs targeted by Upf1-Nos among Bru-repressed mRNAs. Underlying data are in S2 Data. (PDF) [file pbio.3002840.s008.pdf]

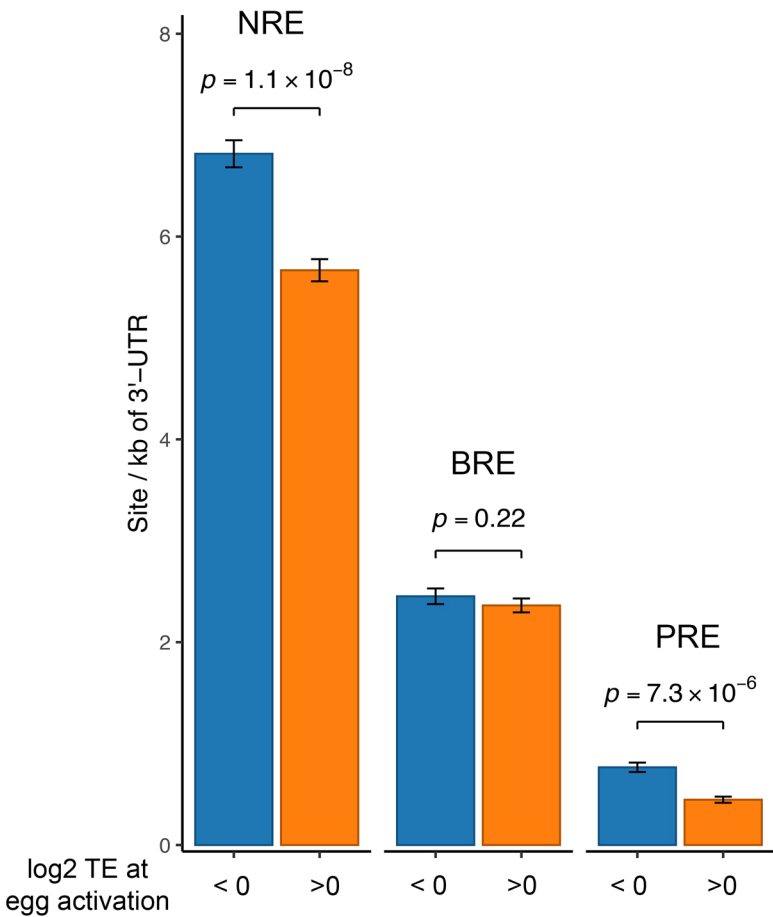

Supplement: S9 Fig — Bar graph showing enrichment in the density (sites/kb of 3′ UTR on the y-axis) of the 3 motifs shown above in translationally repressed mRNAs (negative vs. positive TE), with p-values calculated in the Wilcoxon test. Figure is a LOESS analysis of the data shown in Fig 5C. Underlying data are in S4 Data. (PDF) [file pbio.3002840.s009.pdf]

# Pum-bound mRNAs from Laver et al., 2015

( $p = 0.98$ )

Enrichment 1.2

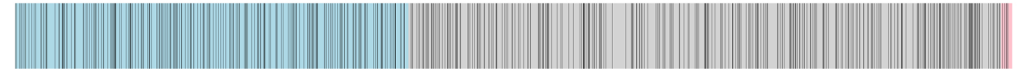

-9.70 -3.89 -2.61 -1.68 -0.97 -0.50 -0.21 -0.02 0.17 0.43 5.36

log<sub>2</sub>(Fold Change) in Upf1-Nos

Supplement: S10 Fig — Barcode plot showing no significant enrichment among mRNAs depleted by Upf1-Nos of mRNAs bound by Pum in 0–3-hour embryonic extracts identified by Laver and colleagues. Zygotically expressed mRNAs in their data were omitted from the analysis. Underlying data are in S2 Data. (PDF) [file pbio.3002840.s010.pdf]

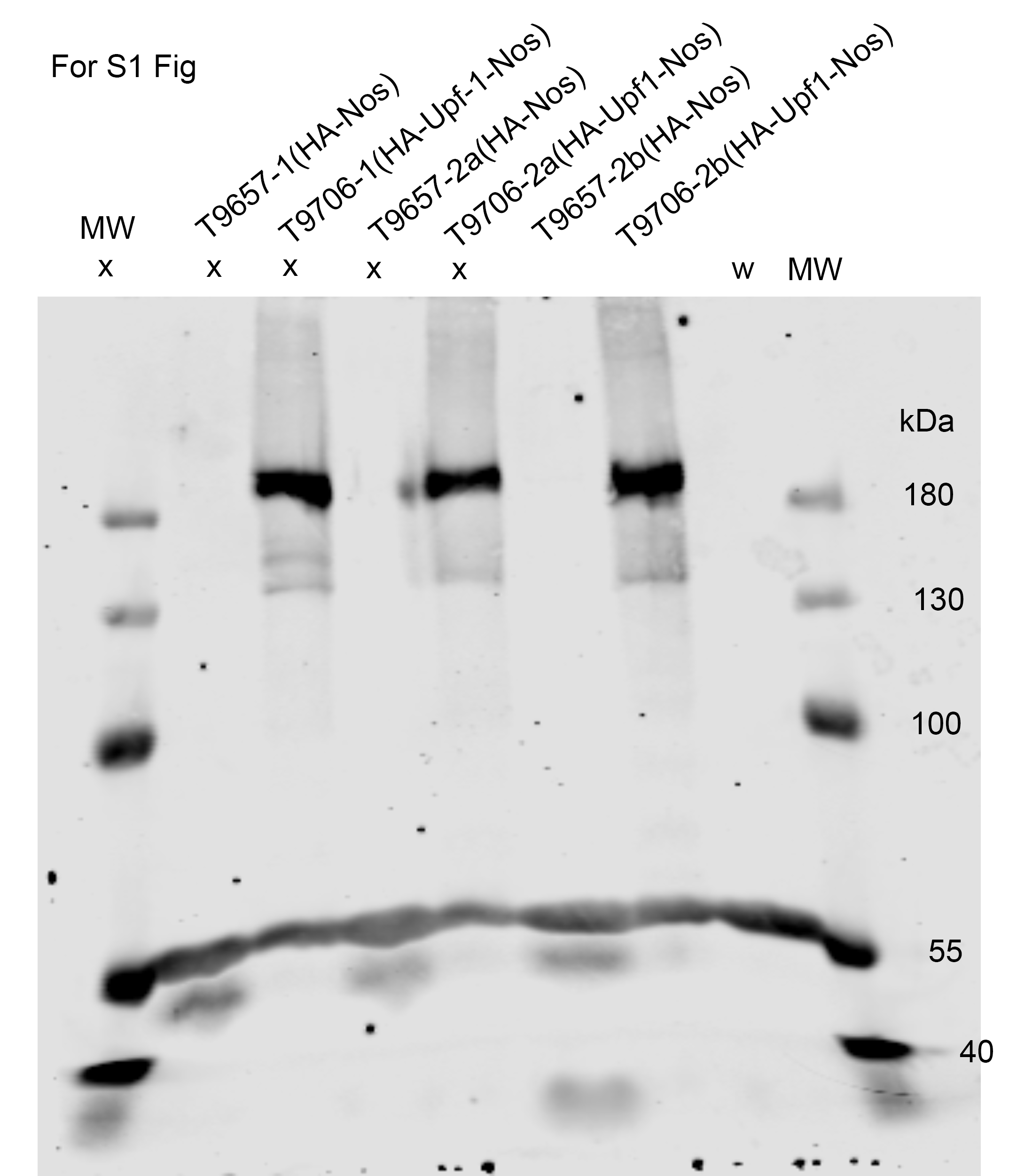

Supplement: S1 Raw Images — (TIF) [file pbio.3002840.s018.tif]
